# Study on Trichothecene and Zearalenone Presence in Romanian Wheat Relative to Weather Conditions

**DOI:** 10.3390/toxins11030163

**Published:** 2019-03-15

**Authors:** Oana Stanciu, Cristina Juan, Houda Berrada, Doina Miere, Felicia Loghin, Jordi Mañes

**Affiliations:** 1 Department of Bromatology, Hygiene, Nutrition, Faculty of Pharmacy, “Iuliu Haţieganu” University of Medicine and Pharmacy, 6 Louis Pasteur, 400349 Cluj-Napoca, Romania; oana.stanciu@umfcluj.ro (O.S.); dmiere@umfcluj.ro (D.M.); 2 Laboratory of Food Chemistry and Toxicology, Faculty of Pharmacy, University of Valencia, Av. Vicent Andrés Estellés s/n, Burjassot, 46100 Valencia, Spain; houda.berrada@uv.es (H.B.); jordi.manes@uv.es (J.M.); 3 Department of Toxicology, Faculty of Pharmacy, “Iuliu Haţieganu” University of Medicine and Pharmacy, 6 Louis Pasteur, 400349 Cluj-Napoca, Romania; floghin@umfcluj.ro

**Keywords:** mycotoxins, GC-MS/MS, cereals, Romania, precipitations, air temperature

## Abstract

To evaluate the influence of weather conditions on mycotoxin presence in wheat, deoxynivalenol (DON), 3-acetyldeoxynivalenol (3AcDON), 15-acetyldeoxynivalenol (15AcDON), fusarenon-X (FUS-X), nivalenol (NIV), HT-2 toxin (HT-2), T-2 toxin (T-2), diacetoxyscirpenol (DAS), neosolaniol (NEO) and zearalenone (ZEN) were evaluated in 102 Romanian wheat samples coming from five wheat growing areas during 2015. Only six mycotoxins were detected, while FUS-X, DAS, NEO and NIV were not present in the wheat samples. Mycotoxin concentrations were correlated with precipitation and temperature values during anthesis and the preharvest period. Overall, the highest frequency was registered for DON, while the lowest frequency was registered for NIV. In the North Muntenia, DON and ZEN registered high frequencies (68% and 16%, respectively). This region was characterized in June and July by medium to high values of rainfall (41–100 mm/month) and normal temperatures (mean of 20.0 °C in June and 24.0 °C in July), suggesting that precipitation levels influence fungi and mycotoxin development to a greater extent compared to the influence of temperature.

## 1. Introduction

Wheat (*Triticum aestivum* L.) has an important contribution to human nutrition, being the basis for a wide variety of bakery products, mainly bread, biscuits, breakfast cereals, pasta, cakes or other [1]. Europe is the second region, after Asia, in terms of both wheat production (249 million tons) and harvested area (58.7 million hectares), while in terms of consumption, Europe is the largest wheat and wheat product consumer (107 kg per capita per year) [2]. 

*Fusarium* species are notable among wheat pathogens, being capable of producing trichothecenes and zearalenone (ZEN) [3,4]. Trichothecenes are a group of tetracyclic sesquiterpenoid substances produced by *Fusarium sporotrichioides*, *F. langsethiae*, *F. graminearum*, *F. culmorum*, *F. poae*, and *F. equiseti* [5,6], including deoxynivalenol (DON), nivalenol (NIV), 3-acetyldeoxynivalenol (3AcDON), 15-acetyldeoxynivalenol (15AcDON), fusarenon-X (FUS-X), HT-2 toxin (HT-2), T-2 toxin (T-2), diacetoxyscirpenol (DAS) and neosolaniol (NEO) [7]. ZEN is a non-steroidal estrogenic mycotoxin produced mostly by *F. graminearum* and *F. culmorum* [6]. According to the available toxicological data concerning carcinogenicity in humans, ZEN, DON, NIV and FUS-X were included by the World Health Organization’s (WHO) International Agency for Research on Cancer (IARC) in the Group 3 [8]. Research on mycotoxin combinatory effects have been initiated over the past years [9], with the co-occurrence of mycotoxins in foodstuffs becoming an important topic for mycotoxin presence and risk assessment studies for both humans and animals [10,11,12].

It is anticipated that climate changes of the planet will produce a warming of the ecosystem, affecting fungal growth, distribution and mycotoxin production in cereals and derivative products and increasing the risks in food safety [13,14,15]. Romania has a continental temperate climate with various influences: oceanic in the central and western regions, continental in the east and Mediterranean in the south [16]. Evaluation of frequency and tendencies of contamination with mycotoxins on the agro-food chain, particularly for cereals, has special importance in the context of climate changes predicted for Romania. These changes include not only the increase of temperature by 3–5 °C and decrease of rainfall in summer, however also terrestrial stilling, seasonal changes in relative air humidity linked with changes in streamflow regime, cloud cover and evapotranspiration [16,17].

*Fusarium* mycotoxin development is dependent on diverse factors, with weather conditions, such as temperature, precipitation and humidity of the surrounding environment, being the most important [11,18]. The environmental conditions that promote *Fusarium* spp. development are moderate temperatures (between 20 and 30 °C) together with high relative humidity (90%), frequent rainfall during and after flowering, extended periods of high moisture and the occurrence of air currents. The regional distribution of mycotoxins also depends on endogenous and exogenous factors that can influence mycotoxin production, e.g., agronomic practices, fungicides used, host resistance and preceding crop [3,19,20].

The presence of mycotoxins in the agro-food chain is considered a food safety and security issue. Considering that, during past years, Romania has been a leader in terms of wheat production in Europe [21], and taking into account its temperate-continental climate, studies on tendencies of mycotoxin contamination are required, more in the context of the climate changes that are predicted for Romania [17]. Therefore, the goals of the present work were: (i) to evaluate the presence of nine trichothecenes (DON, NIV, 3AcDON, 15AcDON, FUS-X, HT-2, T-2, NEO, DAS) and ZEN in Romanian wheat harvested in 2015 using a sensitive gas chromatography tandem mass spectrometry (GS-MS/MS) analytical method; (ii) to assess the influence of the climatic conditions during the grain-growing season on *Fusarium* mycotoxin presence.

## 2. Results

### 2.1. Method Validation and Performance

In mycotoxin analysis, modern generation liquid chromatography tandem mass spectrometry (LC-MS/MS) approaches are the most used, however they also present some disadvantages, including chemical waste [22]. In this study, a gas chromatography - triple quadrupole tandem mass spectrometry (GC-QqQ-MS/MS) method was used to analyze 10 mycotoxins. Even if a derivatization step needs to be added, the preference for this method included the small quantities of reagents used, however more important was the possibility of getting the smallest detection limits for some mycotoxins below the legislated levels of these mycotoxins in cereals, particularly for DON, 3AcDON, 15AcDON and NIV. This step included a liquid-liquid extraction with hexane, an efficient clean-up to eliminate hydrophobic substances that can produce a matrix effect on the ionization.

Concerning the MS/MS, for each compound, two transitions were reached: one for quantification (Q), and another one for confirmation (q). Table 1 shows the parameters of the GC-QqQ-MS/MS method and the results calculated for the method validation. 

The matrix effect (ME) ranged from 64% (ZEN) to 137% (FUS-X). A good linearity was observed, with corresponding correlation coefficients (r^2^) higher than 0.989. The limits of quantification (LOQs) of the mycotoxins analyzed presented high variability and were between 1 μg kg^−1^ (DON) and 20 μg kg^−1^ (NIV and NEO). The accuracy was evaluated for each compound by calculating the recovery values that were between 69% and 127%. Intraday and interday precision values as relative standard deviations (RSDs) were lower than 12% and 19%, respectively. Taking into account our results for the validation of the method, this analysis is sensitive, precise and reproducible. The performance of the method is reflected in the low LOQs and the good recovery rates.

### 2.2. Mycotoxin Occurrence Data in Romanian Wheat During 2015

**Figure 1:**
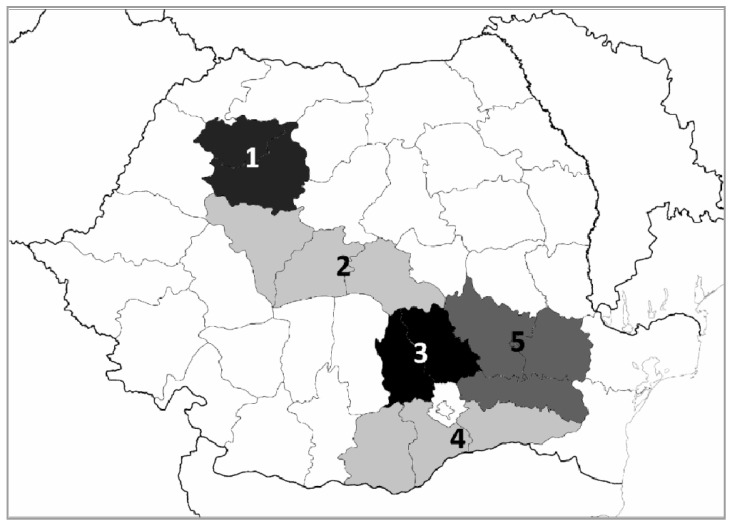
The five Romanian regions included in the study, belonging to different climatic areas: 1. North-West (*n* = 9), 2. Mideast (*n* = 9), 3. North Muntenia (*n* = 31), 4. South Muntenia (*n* = 37), 5. South-East (*n* = 16).

The aim of the present study was to monitor the occurrence of nine trichothecenes and ZEN in 102 wheat samples collected during the 2015 growing season from fields located in five different regions of Romania (Figure 1) with various agroclimatic conditions. 

Results show that 67% (68 samples) of the samples presented detectable levels of at least one mycotoxin: DON, 3AcDON, 15AcDON, NIV, HT-2 and ZEN (Table 2). FUS-X, DAS, NEO and NIV were not detected in the 102 samples analyzed. Most of the positive samples were contaminated with one mycotoxin (48%, 49 samples), followed by the presence of two mycotoxins (11%: DON + ZEN or DON + HT-2), three mycotoxins (4%), four mycotoxins (3%) and five mycotoxins (1%). Three wheat samples exceeded the maximum permitted level (ML) of 100 μg kg^−1^ for ZEN established by the European legislation for unprocessed wheat, with concentrations between 155 and 300 μg kg^−1^ [23].

To the best of the author’s knowledge, recently published data investigating the frequency and levels of both legislated and non-legislated *Fusarium* mycotoxins in unprocessed wheat from Balkan countries with similar climatic conditions as Romania, e.g., Bulgaria, Croatia, Serbia and Slovenia, is rather low. Furthermore, depending on the methods applied for the analysis and their sensitivities, a high variability in interpreting the results could appear. Therefore, a brief revision of the literature was conducted with the goal to introduce insight into the occurrence of *Fusarium* mycotoxins in wheat from Romania during the previous years (Table 3). As can be observed in Table 3, there is only sporadic published data investigating the occurrence of *Fusarium* mycotoxins in wheat from Romania during the last decade. Most of the studies focused on DON and ZEN evaluation, with only three studies including other trichothecenes such as 3AcDON, 15AcDON, NIV, DAS, NEO, HT-2 or T-2 [24,25,26]. The highest frequency percentages were registered for DON, ranging from 19% to 90%, and the highest concentration found (5027 μg kg^−1^) corresponded also to DON in a wheat sample harvested in 2012 [27]. Furthermore, the most used method was the enzyme-linked immunosorbent assay (ELISA), sometimes having low sensitivities reflected within high LODs, and only two studies used multi-class analysis, one by gas chromatography coupled with mass spectrometry (GC/MS), and another one by liquid chromatography tandem mass spectrometry (LC-MS/MS). Hence, sensitive validated multi-mycotoxin methods are recommended to be carried out with the aim to fill the gap concerning mycotoxin evaluation in wheat and its products from Romania.

Concerning the co-occurrence, other authors also remarked that binary combinations of mycotoxins are most often found in cereals. For example, Bryła et al. [34], after analyzing 26 mycotoxins in winter wheat from Poland, concluded that the simultaneous contamination with two mycotoxins was the most frequent (25%) in terms of co-occurrence, with DON currently being found in various combinations and being frequently accompanied by enniatins and/or other emerging mycotoxins. On the other hand, Juan el al. [35] concluded that in Italian wheat from 2013, the most frequently co-contamination occurrence was for three mycotoxins (35%), followed by combinations of two (20%) or four (20%) mycotoxins.

### 2.3. Climate Influence

**Figure 2:**
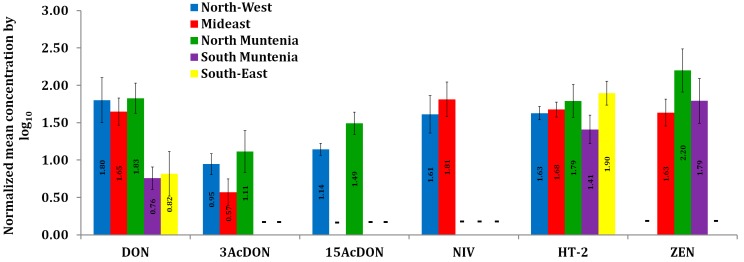
Normalized mean concentrations of mycotoxins in wheat across the five different Romanian regions. Mean concentrations were normalized by Log_10_ (1 + *a*) where *a* is the average concentration of the positive samples expressed in μg kg^−1^. When a mycotoxin was not detected in the wheat samples in a region, no normalized value was calculated and a short horizontal line (-) corresponding to that mycotoxin in that region was used.

The results obtained in the present study indicated various differences in mycotoxin frequency and concentration levels throughout the five regions evaluated. On the other hand, similar trends were observed for the mean concentrations of DON, HT-2 and ZEN in wheat samples from Mideast and North Muntenia (Figure 2). The co-occurrence of mycotoxins was very frequent in the Mideast of Romania (56% of the wheat samples being contaminated with two to five mycotoxins), the North-West (33% of the wheat samples being contaminated with three or four mycotoxins) and the North Muntenia (23% of the wheat samples being contaminated with two to four mycotoxins) (Table 2). Despite the low number of the samples for each region, it can be remarked that in the North-West of Romania, there is a predisposition for simultaneous contamination with three or four mycotoxins, while in the North and South Muntenia, there is a tendency for contamination with two mycotoxins. Concerning DON, that was the most frequent mycotoxin and the contamination incidence decreased as following: North-West (100%) > Mideast (89%) > North Muntenia (68%) > South Muntenia (43%) > South-East (38%). The Pearson coefficient indicated a strong positive linear relationship (*y* = 2.8679*x* + 4.5957; r^2^ = 0.784) between DON and ZEN concentrations.

Highly significant correlations between DON and ZEN, as well as between DON and NIV, were also observed by Vogelgsang et al. [36] after an eight year monitoring study of various mycotoxins in wheat from Switzerland. These results underline the potential of *F. graminearum* to produce DON and ZEN simultaneously within a single isolate under the influence of various factors related to the crop or the environment [7,36].

The highest mean concentrations for DON (66.3 μg kg^−1^), 3AcDON (12 μg kg^−1^), 15AcDON (30 μg kg^−1^), HT-2 (60.8 μg kg^−1^) and ZEN (157 μg kg^−1^) were found in the same region, North Muntenia. Interestingly, the maximum levels of DON (955 μg kg^−1^), 3AcDON (12 μg kg^−1^), 15AcDON (30 μg kg^−1^) and ZEN (300 μg kg^−1^) were found in the same sample from this region, suggesting that there may be a close link between the concentrations of DON, its acetylated derivatives and ZEN.

**Figure 3:**
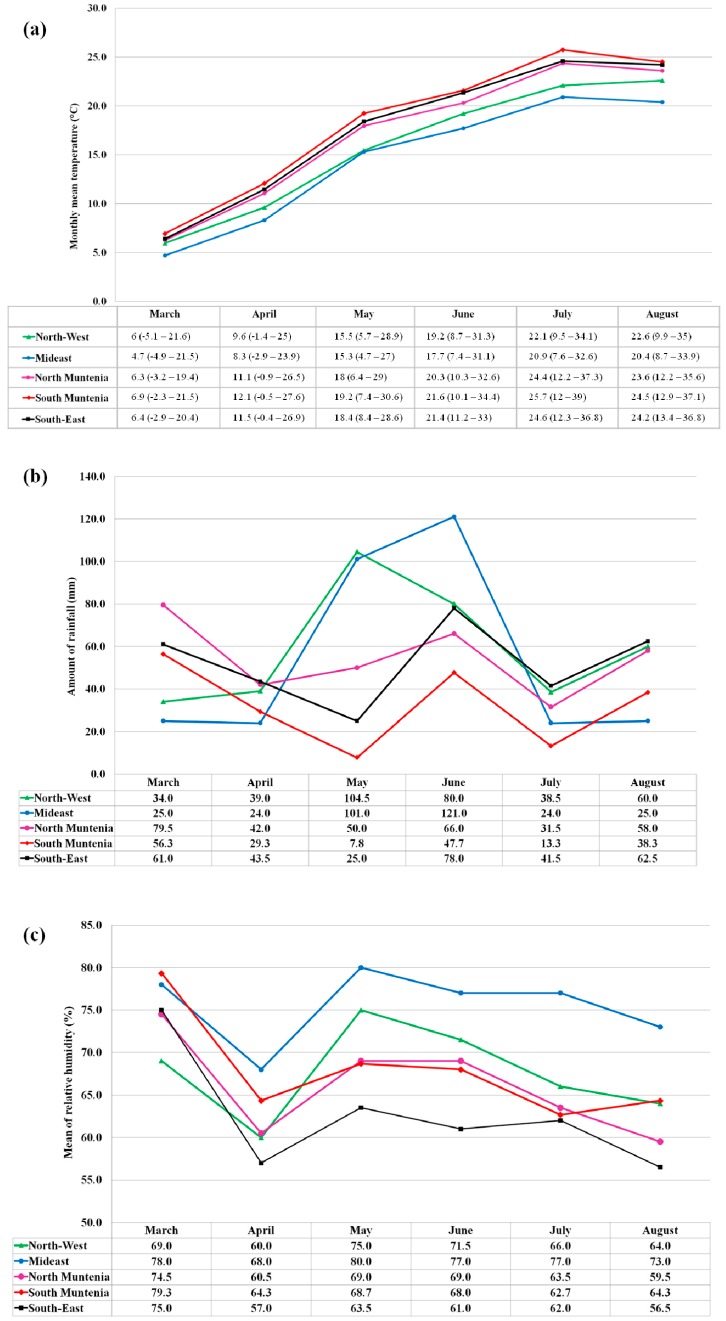
Evolution of (**a**) monthly mean temperatures; (**b**) monthly amount of rainfall; and (**c**) mean of relative humidity in Romania during 2015 (April–August) [37].

Regarding the weather conditions in Romania during the 2015 year (Figure 3), some particularities were observed. The region with the highest levels of *Fusarium* mycotoxins in wheat (DON, 3AcDON, 15AcDON, HT-2 and ZEN), the North Muntenia, was characterized in May (anthesis period) by moderate quantities of rainfall (mean quantity of 50 mm), medium air humidity (69%) and normal average temperatures (mean of 18 °C), and in June and July (from late anthesis to yield formation) across this region, medium to high quantities of precipitation (between 51–100 mm, with a mean of 66 mm and between 21–75 mm, with a mean of 31.5 mm, respectively), medium air humidity (69% and 63.5%, respectively) and normal temperatures (mean of 20.3 °C and 24.4 °C, respectively) were registered. It must be mentioned that after analyzing climatic details, we remarked that the North Muntenia region registered the highest number of rainy days during May and June 2015 (14 from 31 days and 15 from 30 days, respectively) which can suggest that high moisture could be maintained in wheat grains, influencing the water activity and, consequently, the development of molds and mycotoxins. Furthermore, the second region in terms of mycotoxin presence in wheat samples, the Mideast of Romania, registered high to abundant quantities of rainfall (mean of 101 mm) in May, while in June, abundant to excessive quantities of precipitation (between 101–175 mm, with a mean of 121 mm) were reported. On the other hand, this region was characterized by the lowest monthly average temperatures and the highest air relative humidity percentages compared to the other regions from April to June 2015. Also, the North-West Romania, remarked by the highest incidence of DON, was the region that recorded the rainiest month of May during 2015 (with quantities between 126–200 mm and a monthly average of 104.5 mm), coupled with normal temperatures (mean of 15.5 °C) and normal humidity (75%) [37].

Environment parameters, particularly temperature, precipitation and relative air humidity, are relevant factors for fungal infection, mycotoxin production and survival. It should be mentioned that at regional the level, mycotoxin distribution depends on various factors such as agronomic practices, fungicides used, host resistance and preceding crop [3,19,20]. Even if the ANOVA single factor test did not show a statistically significant correlation between mycotoxin content and climatic parameters, some trend can be easily observed. A prolonged rainy weather during the earing phase, anthesis, dough formation and filling (beginning of May to the end of June) could favor high moisture for the wheat crops, consequently influencing fungi development and mycotoxin production, particularly DON and ZEN, and, depending on various factors, also HT-2. Moreover, it can be affirmed that the quantity of rainfall (especially during May) possesses more capacity to influence mycotoxin content in wheat, followed by air relative humidity and air temperature.

The results from the present study revealed the possible relationship between the presence of *Fusarium* mycotoxins, particularly DON, HT-2 and ZEN, in wheat and the climatic parameters during the 2015 harvest season. In a previous study, published by us, 35 wheat samples that were harvested during the 2014 season from four Romanian counties were analyzed to evaluate trichothecene, ZEN and emerging mycotoxin presence, and the results revealed statistically significant differences (*p* < 0.05) for DON and ZEN [26]. Interestingly, the highest frequencies and levels of DON and ZEN were registered in the North Muntenia region and the Mideast of Romania in both the 2014 and 2015 harvest years. During 2014, for these two regions, a monthly precipitation deviation against the multiannual mean between 51% and 75% was reported in May and July, coupled with mean temperatures with 2–4 °C less than normal, very wet days and high humidity [26], while during 2015, as it is reported in the present study, these regions are characterized by the highest number of rainy days during May and June, coupled with abundant to excessive quantities of precipitation. Taking these results into account, the present study comes to emphasize the idea that the contamination with mycotoxins could be considered to be local, with the meteorological factors being able to have a decisive influence on this.

Until now, the Romania studies performed on mycotoxin presence in wheat were focused mostly on DON (Table 3). For example, Gagiu et al. [27], after evaluating DON levels in 1754 cereal samples from Romania (common wheat, durum wheat, triticale and wheat) and the meteorological, hydrological and geographical parameters, stated that the North-West of Romania presents a possible risk for DON contamination. The same conclusion was also reached by Alexa et al. [32] after analyzing 52 wheat samples collected from Western Romania during two consecutive harvest years. The newest data published about mycotoxins in Romanian wheat concluded that post-harvest contamination with DON and ZEN has a local character, with the Southern part of Romania being the area of interest due to its semi-arid temperate continental climatic conditions [38].

Somewhat similar ideas were presented by Vogelgsang et al. [36], who mentioned that mycotoxins content could be partially influenced by the weather variables, with other factors also having a strong influence on *Fusarium* production and mycotoxin contamination. The prevailing temperature and moisture immediately before and during anthesis possess a strong effect on both maturation of the perithecia (temperature between 20 and 25 °C and relative humidity higher than 85%) and discharge of ascospores of *Fusarium graminearum* (optimum temperature of 21 °C and relative humidity of 100%), leading to infection and successive contamination with DON and ZEN and, depending on the chemotype, also with NIV [36].

Our results are also in accordance with other studies in countries with a similar climate to Romania. A multi-mycotoxin analysis was performed on 54 wheat samples from different regions of Serbia and significant differences were observed between northern and southern regions within the same year, attributed primarily to the differences in climate conditions and, consequently, in the period of collection. The southern Serbian regions where mycotoxins were not detected in wheat samples were characterized by a specific microclimate with a very low amount of precipitation, while in the northern part of Serbia where mycotoxins were detected, particularly DON (ranging from 41 to 309 μg kg^−1^), precipitation amounts of up to seven times higher were recorded [39].

In a Polish study [34], the presence of 26 mycotoxins in 99 wheat samples from five regions of Poland was monitored. After analyzing the mycotoxin levels and the trends in the prevalence of temperature and rainfall, it was found that the most contaminated wheat samples belonged to the south-eastern regions of Poland where the greatest rainfall and temperature values were recorded during the wheat earing stage and flowering period, compared with the regions located in the North and West of Poland, where lower temperature and lower air humidity limited the mycotoxin biosynthesis. A recent study, published by the same group of research, strongly concluded that the levels of NIV, DON and DON-3-Glucoside depend on weather conditions prevailing in any given growing season at any given geographic position of the cultivation area [40].

An interesting study that evaluated the correlation between mycotoxins in wheat and weather parameters was recently published by Vogelgsang et al. [36]. In total, 686 wheat grain samples from nine different climate regions of Switzerland were analyzed between 2007 and 2014. Results demonstrated that the monitoring year had a highly significant effect on both the DON contamination rate and the average content, however no effect was observed on ZEN or NIV. Statistically, a slightly low variation of DON contents was explained by the climate region and no effect of the climate region was observed on the contents of ZEN or NIV.

For other regions, a research performed by Alkadri et al. [41] indicated a lower incidence of *Fusarium* mycotoxins (DON, 3AcDON, 15AcDON, HT-2, T-2, NIV and ZEN) in Syrian wheat samples compared to the Italian ones, although both the countries are in the Mediterranean area. The authors explained this diversity through climatic conditions. Syria has an arid climate, very hot in the summer and cold in winter, whereas the climate of Italy is mainly temperate and slightly varies according to the areas; the northern Italian regions have warm humid summers compared with the southern part, thus the presence of *Fusarium* spp. and the correspondent mycotoxins is more abundant in the northern regions.

Occurrence of trichothecenes and ZEN in wheat is considered a typical agricultural issue in temperate regions where weather conditions are favorable for *F. graminearum* and *F. culmorum* growth and related mycotoxin production [20]. A significant increase in fungal attacks on wheat has been observed worldwide, with climate changes influencing the occurrence of molds in cereals and the development of mycotoxins [42]. Changes in agroclimatic conditions directly affect fungal populations and related mycotoxins and also have indirect impacts on mycotoxin contamination, such as increased drought stress, insect damage of the plant and modifications in crop phenology [15]. This negative influence of the anthropogenic forced climate change on mycotoxin presence was observed not only in wheat, however also in other crops such as barley, maize, soy, coffee and grapes and, consequently, in beer, wine, soybean and maize products [43,44,45].

Studies on unprocessed food material, such as wheat, are important due to their contribution to risk assessment investigations. It is known that wet weather can sometimes delay the ripening and harvesting periods of wheat crops. Moreover, it is acknowledged that mycotoxins are mostly situated in the outer layers of the grain, thus high fiber or bran based products can present the same or higher concentrations of mycotoxins compared with the raw unprocessed material. A recent study showed that DON and ZEN concentrations for the mill fractions and the cleaned grains were all in agreement with one another, concluding that wet weather during wheat maturation and harvesting not only impacts *Fusarium* mycotoxin levels, however it can also modify the distribution of some compounds from this group within the mill fractions [46].

## 3. Conclusions

A GC-MS/MS method was validated for the determination of nine trichothecenes and ZEN in wheat, with good accuracy and high sensitivity. The efficiency of the method was supported by evaluating the presence of the 10 mycotoxins in 102 Romanian wheat samples collected during the 2015 harvest year. Only DON, 3AcDON, 15AcDON, NIV, HT-2 and ZEN were detected in the analyzed samples, with the simultaneous presence of the four type A trichothecenes (DON, 3AcDON, 15AcDON, NIV) being important for risk assessment due to the same metabolic pathway and possible synergic toxic effects of these mycotoxins. Interestingly, neither DAS nor FUS-X, NEO and T-2 were detected in the wheat samples that were evaluated. The absence of DAS in these wheat samples could be explained by the possibility that this compound exists in the form of DAS-glucoside or because this mycotoxin is more frequently found in cereal-based dishes, not in raw cereals. Data obtained was linked with the weather parameters in the growing region.

Based on the present results and the available literature on this topic, it can be stated that extremely phenomenal—rainy periods at the end of flowering, drought during grain formation or high moisture in the late preharvest period—are favorable, particularly for DON, HT-2 and ZEN presence and the simultaneous occurrence of DON and ZEN in wheat. Even so, the results should be interpreted with high caution because mycotoxin presence is not justified by one single pattern, geographic position and agricultural practices, and also, the weather parameters all have various effects on the presence of *Fusarium* head blight, particularly for the main species *Fusarium graminearum* and its major micotoxins.

These observations become important in the context of the predicted climate changes that could also affect fungi development and mycotoxin production. This comprehensive evaluation was done for the first time in Romania (which is a dominant country in terms of wheat production in the Balkan area) for both legislated and non-legislated mycotoxins, using a highly sensitive analytical method. Therefore, the present study can contribute to the effort to reduce fungi and mycotoxin attacks in wheat, and it can represent an important step for the mitigation strategies and the hazard analysis and critical control points (HACCP) monitoring process. The analysis of mycotoxins and the evaluation of the agroclimatic parameters should be used as control points, helping to make an effective and useful control. To protect human health, continuous studies concerning mycotoxin presence in wheat associated with the environmental conditions, particularly in the Balkan area, are required. Furthermore, a detailed multivariate study including environmental, geographic and agricultural factors will help to evaluate the trend of *Fusarium* mycotoxin development.

## 4. Materials and Methods

### 4.1. Chemicals and Reagents

Reagents were purchased as following: acetonitrile and hexane from Merck KGaA (Darmstadt, Germany), methanol LC-MS/MS grade (≥99.9% purity) from VWR International Eurolab (Barcelona, Spain), the derivatisation reagent BSA (*N*,*O*-bis(trimethylsilyl)acetamide) + TMCS (trimethylchlorosilane) + TMSI (*N*-trimethylsilyimidazole) (3:2:3) from Supelco (Bellefonte, PA, USA), sodium dihydrogen phosphate and disodium hydrogen phosphate (needed to form the phosphate buffer) from PanReac AppliChem (Castellar del Vallés, Spain).

Deionized water (<10 MΩ cm^−1^ resistivity) was constructed in the laboratory using a Milli-Q SP^®^ Reagent Water System (Millipore, Bedford, MA, USA).

The extract samples were filtered using Whatman no. 4 filter papers (Maidstone, UK). Polypropylene syringes (2 mL) and nylon filters (13 mm diameter, 0.22 μm pore size) were supplied by Análisis Vínicos S.L. (Tomelloso, Spain).

The certified standards of DON, NIV, 3AcDON, 15AcDON, FUS-X, HT-2, T-2, NEO, DAS and ZEN were purchased from Sigma Aldrich (Madrid, Spain). The individual stock solutions of all mycotoxins were prepared in acetonitrile at a concentration of 1000 μg mL^−1^. A combined standard solution in the same solvent at a concentration of 500 μg mL^−1^ was prepared. This solution was used to prepare the calibration curves, matrix matched calibration curves and for the method validation. Matrix-matched calibration curves for mycotoxin quantification were constructed using successive dilutions from 400 to 10 μg kg^−1^. For the quantification in wheat samples, matrix-matched calibration curves were used. The solutions were kept in glass-stoppered bottles which were protected from light at −20 °C.

### 4.2. Sampling

A total of 102 whole unprocessed wheat samples were collected during the 2015 harvest season from five different Romanian regions with the aim to investigate mycotoxin presence. The criterion used to include a wheat sample in the present study was that the wheat sample must be a variety (organic or conventionally cultivated) produced for human consumption. The number of wheat samples for each region was influenced by the prevalence of cultivation of wheat in that area, which was directly correlated with the geographic conditions [16].

Information on the growing area (county and city), cultivation and harvest period was considered. Sampling was performed according to the European Union (EU) guidelines [47]. After homogenization, samples were packed in plastic bags and kept at −20 °C and protected from light. Before the analysis, for all the samples, subsamples of 300 g were milled with a blender and divided into three bulks of 100 g each. The experiments were performed in triplicate.

### 4.3. Sample Preparation

#### 4.3.1. Extraction

Briefly, 2 g of each sample were weighed, placed into 50 mL polytetrafluoroethylene (PTFE) centrifuge tubes and extracted as mentioned in the method of Stanciu et al. [26]. After this, 5 mL of supernatant were placed in 15 mL PTFE centrifuge tubes and were evaporated to dryness at a temperature of 35 °C with a soft stream of nitrogen with the aid of a multi-sample Turbovap LV Evaporator (Zymark, Hoptkinton, MA, USA). The residue was reconstituted with 1 mL of a mixture of methanol and water (70:30, *v*/*v*), filtered through a syringe nylon filter and 200 μL of filtrate were dried under nitrogen flow.

#### 4.3.2. Derivatisation

Over the dry extract, 50 μL of BSA reagent were added and the sample was allowed to stand for 30 min at room temperature. The derivatised sample was diluted to 200 μL with hexane and was mixed thoroughly on a vortex for 30 s. After this, the hexane was washed using 1 mL of phosphate buffer (60 mM, pH 7) and was agitated using the vortex until the upper layer was clear. Finally, the upper layer was moved to an autosampler vial for chromatographic analysis.

### 4.4. GC-QqQ-MS/MS

For the detection of the 10 mycotoxins, a GC system Agilent 7890A coupled with an Agilent 7000A triple quadrupole mass spectrometer and an Agilent 7693 autosampler (Agilent Technologies, Palo Alto, CA, USA) were used.

Quantitative data were acquired at a selection reaction monitoring (SRM) mode and the mass spectrometer was managed in an electron ionization mode (70 eV). The temperature of the transfer line was 280 °C, while the source temperature was 230 °C. For the MS/MS system, the collision gas was nitrogen with a flow of 1.5 mL min^−1^, while helium was used as the quenching gas with a flow of 2.25 mL min^−1^, both having a purity of minimum 99.999% and being supplied by Carburos Metálicos S.L. (Barcelona, Spain). To acquire and process the data, the Agilent Masshunter version B.04.00 software was used.

For the separation of the analytes, a HP-5MS 30 m × 0.25 mm × 0.25 μm capillary column was used. A volume of 1 μL of the cleaned extract was injected in splitless mode into a programmable temperature vaporization inlet (150 °C for 0.1 min then 250 °C for 5 min) setting helium as the carrier gas, at a fixed pressure of 20.3 psi. The oven temperature was initially set at 80 °C and was then was increased to 245 °C at 60 °C min^−1^ (3 min of hold time), continuing to 260 °C at 3 °C min^−1^ and to 270 °C at 10 °C min^−1^ (held for 10 min).

### 4.5. Method Validation

The method was validated evaluating linearity, accuracy, repeatability (intraday and interday precision) and sensitivity, according to the EU Commission Decision [48]. The criterion for confirmation of positive findings was: comparison of peak area ratios for quantification (Q) and confirmation (q) transitions with that of the reference standard; peak ratio of the confirmation transition against quantification one; accordance with the retention times.

A blank of wheat, previous analyzed and negative for the mycotoxins included in this study, was used for the method validation. Matrix-matched calibration curves were constructed at concentration levels between 10 and 400 μg kg^−1^. Matrix effect (ME) was evaluated for each compound, comparing the slope of the standard calibration curve (a_standard_) with that of the matrix-matched calibration curve (a_matrix_) for the same concentration levels. Limit of detection (LOD) and limit of quantification (LOQ) were estimated using an extract of the blank for a signal-to-noise ratio (S/N) of ≥ 3 and ≥ 10, respectively, from chromatograms of samples spiked at the lowest level validated. Accuracy was estimated through recovery studies carried out by spiking blank wheat at three concentration levels: low (LOQs), medium (two times more the LOQs) and high (10 times more the LOQs). Precision was evaluated using the relative standard deviation (RSD) of the results obtained during the same day (intraday) and on three different days (interday) by the repeated analysis three times at the three spiked levels.

### 4.6. Climate Conditions

Data about climatic conditions was extracted from the database of the Romanian National Meteorological Administration (Meteo Romania) [37]. Gridded data spatially interpolated was used to obtain the mean precipitation and mean air temperature from April to July, covering the two important periods in the growing process of wheat: anthesis (months of April and May) and the preharvest period (months of June and July).

### 4.7. Statistical Analysis of the Data

Results are reported as the mean ± standard deviation. The correlation between DON and ZEN levels was performed by the Pearson Correlation test, and the correlation between mycotoxin concentration and climatic parameters was evaluated by applying ANOVA single factor test. Statistical procedures were performed using SPSS software, version 22.0 (IBM Corp, Armonk, NY, USA).

## Figures and Tables

**Table 1:** Chromatographic and analytical parameters for mycotoxin detection by GC-MS/MS: retention time (Rt); quantification (Q) and confirmation (q) transitions; collision energy (CE); dwell time (Dt); limits of detection (LOD) and quantification (LOQ); recovery at three spiked concentration levels; interday relative standard deviation (RSD); matrix effect (ME); linearity expressed as a correlation coefficient (r^2^).

Analyte	Rt (min)	SRM (Selection Reaction Monitoring) Transitions (*m*/*z*)	CE (eV)	Dt (ms)	LOD (μg kg^−1^)	LOQ (μg kg^−1^)	Recovery (RSD) (%)						ME ± RSD (%)	Linearity (r^2^)
							LOQ		2 LOQ		10 LOQ			
DON	8.45	392 > 259 ^Q^	10	25	0.5	1	100	(7)	95	(1)	127	(2)	85 ± 9	0.994
		407 > 197 ^q^	10	25										
3AcDON	9.45	392 > 287 ^Q^	10	25	1.25	2.5	84	(17)	85	(4)	117	(15)	133 ± 7	0.995
		467 > 147 ^q^	5	35										
15AcDON	9.65	392 > 217 ^Q^	20	35	2.5	5	87	(1)	82	(6)	108	(11)	102 ± 6	0.996
		392 > 184 ^q^	20	35										
FUS-X	9.55	450 > 26 ^Q^	10	35	2.5	5	79	(4)	73	(3)	97	(7)	137 ± 4	0.998
		450 > 245 ^q^	20	35										
DAS	9.56	350 > 229 ^Q^	15	35	7.5	15	83	(16)	77	(11)	118	(10)	66 ± 6	0.989
		378 > 124 ^q^	10	25										
NIV	9.90	289 > 73 ^Q^	15	35	10	20	78	(3)	88	(1)	117	(5)	97 ± 9	0.994
		379 > 73 ^q^	15	35										
NEO	11.30	252 > 195 ^Q^	10	25	10	20	75	(4)	80	(1)	86	(9)	129 ± 3	0.999
		252 > 167 ^q^	15	35										
HT-2	14.40	347 > 185 ^Q^	10	25	7.5	15	100	(19)	84	(5)	84	(7)	111 ± 6	0.989
		347 > 157 ^q^	10	25										
T-2	14.45	350 > 229 ^Q^	10	25	2.5	5	103	(1)	107	(1)	113	(5)	101 ± 1	0.989
		350 > 259 ^q^	15	35										
ZEN	15.46	462 > 151 ^Q^	20	25	5	10	69	(8)	106	(8)	97	(3)	64 ± 4	0.999
		462 > 333 ^q^	20	25										

**Table 2:** Incidence and concentration levels of the mycotoxins detected in the Romanian wheat samples collected during the 2015 harvest year.

Region	Parameter	Mycotoxin						Co-Occurrence	
		DON	3AcDON	15AcDON	NIV	HT-2	ZEN	(%)	Combinations (Number of Samples)
North-West (*n* = 9)	Frequency (%)	100	22	22	11	11	11	3/9 (33)	DON + 3AcDON + 15AcDON (2)
	Mean (μg kg^−1^)	62.4	7.83	12.9	40	41.5	n.q.		DON + NIV + HT-2 + ZEN (1)
	Range (μg kg^−1^)	2.3–323	7.83	8.9–16.9	40	41.5	n.q.		
Mideast (*n* = 9)	Frequency (%)	89	22	11	22	33	33	5/9 (56)	DON + ZEN (1)
	Mean (μg kg^−1^)	43.5	2.7	n.q.	63.8	46.4	42.1		DON + HT-2 (1)
	Range (μg kg^−1^)	1.3–129	2.72	n.q.	63.3–64.3	35.2–67.4	23.8–77.7		DON + NIV + HT-2 (1)
									DON + 3AcDON + 15AcDON + ZEN (1)
									DON + 3AcDON + NIV+HT-2 + ZEN (1)
North Muntenia (*n* = 31)	Frequency (%)	68	3	6	0	10	16	7/31 (23)	DON + ZEN (3)
	Mean (μg kg^−1^)	66.3	12	30	n.q.	60.8	157		DON + HT-2 (2)
	Range (μg kg^−1^)	1.1–955	12	30	n.q.	31.1–98.5	12.4–300		DON + 15AcDON + HT-2 (1)
									DON + 3AcDON + 15AcDON + ZEN (1)
South Muntenia (*n* = 37)	Frequency (%)	43	0	0	0	8	19	4/37 (11)	DON + ZEN (3)
	Mean (μg kg^−1^)	4.7	n.q.	n.q.	n.q.	24.7	60.9		DON + HT-2 (1)
	Range (μg kg^−1^)	1.2–16.4	n.q.	n.q.	n.q.	24.7	11.7–155		
South-East (*n* = 16)	Frequency (%)	38	0	0	0	6	0	0/16 (0)	-
	Mean (μg kg^−1^)	5.5	n.q.	n.q.	n.q.	77.6	n.q.		
	Range (μg kg^−1^)	1.1–11	n.q.	n.q.	n.q.	77.6	n.q.		
Overall (*n* = 102)	Incidence	60	5	5	3	11	16	19/102 (19)	DON + ZEN (7)
	LOD–LOQ	11	2	2	0	2	5		DON + HT-2 (4)
	Frequency (%)	59	5	5	3	11	16		DON + 3AcDON + 15AcDON (2)
	Mean (μg kg^−1^)	44.3	7.5	18.6	55.9	51.7	90.7		DON + 15AcDON + HT-2 (1)
	Range (μg kg^−1^)	1.1–955	2.7–12	8.9–30	40–64.3	24.7–98.5	11.7–300		DON + NIV + HT-2 (1)
	ML (μg kg^−1^)	1750	n.a.	n.a.	n.a.	100 *	100		DON + 3AcDON + 15AcDON + ZEN (2)
									DON + NIV + HT-2 + ZEN (1)
									DON + 3AcDON + NIV + HT-2 + ZEN (1)

Co-occurrence: number of samples presenting levels ≥ limit of detection (LOD) for at least two mycotoxins/total samples from that region (the percentage of samples presenting levels ≥ LOD for at least two mycotoxins/total samples from that region); Frequency: the percentage of samples ≥ LOD/total samples; Incidence: number of samples ≥ LOD; LOD–LOQ: number of samples ≥ LOD and ≤ limit of quantification (LOQ); Mean: average of the positive samples; ML: maximum permitted level established by the European regulations for unprocessed wheat [23,28]; *: ML recommended for the sum of HT-2 and T-2 [28]; n.a.: data not available; n.q.: not quantified because no sample was ≥ LOQ.

**Table 3:** *Fusarium* mycotoxin occurrence and levels in wheat in recent surveys in Romania.

Year	Method	Mycotoxins	LOD (μg kg^−1^)	N	Frequency (%)	Range (μg kg^−1^)	Ref.
n.a.	GC/MS	DON	n.a.	42	90	21–3395	[25]
		15AcDON			36	6–99	
		NIV			2	max. 30	
		DAS			2	max. 19	
		HT-2			50	3–18	
		T-2			2	max. 7	
2008	ELISA	DON	n.a.	40	43	max. 95.7	[29]
		ZEN			10	max. 5.5	
2009	ELISA	DON	n.a.	12	83	6.1–154.3	[30]
		ZEN			50	36.7–67.3	
2009	ELISA	T-2	n.a.	2	100	0.8–1.0	[24]
2008–2010	ELISA	ZEN	n.a.	20	10	0.88–3.6	[31]
2010	ELISA	DON	110	26	73	294–3390	[32]
		ZEN	22.7		69	37.6–1000	
2011	ELISA	DON	110	26	19	254–1440	[32]
		ZEN	22.7		77	28–105.6	
2012	ELISA	DON	18.5	831	65	<18.5–5027	[27]
2013	ELISA	DON	18.5	923	53	<18.5–3602	[27]
2014	ELISA	ZEN	n.a.	336	5	17–80	[33]
2014	LC-MS/MS	DON	20	31	26	110–1787	[26]
		3AcDON	20		0	n.q.	
		15AcDON	150		0	n.q.	
		NIV	150		0	n.q.	
		DAS	30		0	n.q.	
		NEO	7		0	n.q.	
		HT-2	50		0	n.q.	
		T-2	75		0	n.q.	
		ZEN	20		13	327–1135	
2015	ELISA	DON	18.5	4	50	<18.5–964	[17]

LOD: limit of detection reached by the method; Frequency: the percentage of samples ≥ LOD/total samples; N: number of samples; n.a.: data not available; n.q.: not quantified because no sample was ≥ LOD.
